# Examining the association between oncology drug clinical benefit and the time to public reimbursement

**DOI:** 10.1002/cam4.4455

**Published:** 2021-12-01

**Authors:** Sasha Thomson, Louis Everest, Noah Witzke, Tina Jiao, Seanthel Delos Santos, Vivian Nguyen, Matthew C. Cheung, Kelvin K. W. Chan

**Affiliations:** ^1^ Evaluative Clinical Sciences Odette Cancer Centre Research Program Sunnybrook Research Institute Toronto Ontario Canada; ^2^ Odette Cancer Centre Sunnybrook Health Sciences Centre Toronto Ontario Canada; ^3^ Department of Medicine University of Toronto Toronto Ontario Canada; ^4^ Canadian Centre for Applied Research in Cancer Control Toronto Ontario Canada

**Keywords:** clinical benefit, oncology drugs, pCODR, prioritization, reimbursement

## Abstract

**Background:**

We examined if oncology drug indications with high clinical benefit, as measured by the American Society of Clinical Oncology Value Framework (ASCO‐VF) and European Society for Medical Oncology Magnitude of Clinical Benefit Scale (ESMO‐MCBS), received public reimbursement status faster than those with lower clinical benefit from the time of pan‐Canadian Oncology Drug Review (pCODR) recommendation.

**Methods:**

Oncology drug indications submitted to pCODR between July 2011 and October 2018 were examined. Included indications had a regulatory approval date, completed the pCODR review process, received a positive pCODR recommendation, and been funded by at least one province. Trials cited for clinical efficacy were used to determine the clinical benefit (per ASCO‐VF and ESMO‐MCBS) of drug indications.

**Results:**

Eighty‐four indications were identified, yielding 65 ASCO‐VF and 50 ESMO‐MCBS scores. The mean ASCO‐VF and ESMO‐MCBS scores were 44.9 (SD = 21.1) and 3.3 (SD = 1.0), respectively. The mean time to provincial reimbursement from pCODR recommendation was 13.2 months (SD = 9.3 months). Higher ASCO‐VF and ESMO‐MCBS scores had low correlation with shorter time to reimbursement, (*ρ* = −0.21) and (*ρ* = 0.24), respectively. In the multivariable analyses, ASCO‐VF (*p* = 0.40) and ESMO‐MCBS (*p* = 0.31) scores were not significantly associated with time to reimbursement. Province and year of pCODR recommendation were associated with time to reimbursement in both ASCO and ESMO models.

**Conclusions:**

Oncology drug indications with higher clinical benefit do not appear to be reimbursed faster than those with low clinical benefit. This suggests the need to prioritize oncology drug indications based on clinical benefit to ensure quicker access to oncology drugs with the greatest benefits.

## Graphical abstract

This article examined if oncology drug indications with high clinical benefit, as measured by the American Society of Clinical Oncology Value Framework (ASCO‐VF) and European Society for Medical Oncology Magnitude of Clinical Benefit Scale (ESMO‐MCBS), received public reimbursement status faster than those with lower clinical benefit from the time of pan‐Canadian Oncology Drug Review (pCODR) recommendation. It was found that oncology drug indications with high clinical benefit were not significantly associated with shorter time to public reimbursement. These findings suggest the need to implement a transparent system of prioritization to ensure quicker access to oncology drugs with the greatest benefits.

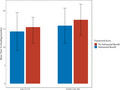

## 1 INTRODUCTION

In order to improve the quality of health care services, the Institute of Medicine (IOM) has recommended that health care organizations, professional groups, and private and public purchasers pursue aims of effectiveness and timeliness.^1^ The aim of effectiveness has been described as providing care that has demonstrated evidence of improved outcomes over existing alternatives.^1^ The aim of timeliness has been described as reducing wait times and potentially harmful delays for those who receive and provide care.^1^ Thus, in the pursuit of both effective and timely health care, patients should ideally have faster access to treatments with the potential for greatest benefits.

The United States (US) Food and Drug Administration (FDA) and Health Canada have both created pathways intended to expedite the regulatory approval of new drugs that have the potential to provide high clinical benefit.^2^, ^3^ The FDA’s breakthrough therapy program grants accelerated approval to drugs that treat serious conditions and have preliminary clinical evidence indicating it may demonstrate substantial improvement over existing treatment options.^2^ Similarly, Health Canada's Notice of Compliance with Conditions (NOC/c) policy provides earlier access to promising new drugs that treat serious conditions for which no drug is currently marketed or for which increased efficacy/decreased risk is demonstrated.^3^ While these designated drugs have been associated with reduced approval times, they have failed to display evidence of significant therapeutic advantage over existing drugs.^4^, ^5^, ^6^

Prioritization based on clinical benefit is equally important for the public reimbursement of drugs. In Canada, the pan‐Canadian Oncology Drug Review (pCODR) is a health technology assessment collaborative initiative that conducts evidence‐based reviews of oncology drugs, providing non‐binding reimbursement recommendations to the provinces and territories (with the exception of Quebec).^7^ Oncology drug indications are submitted to the pCODR by pharmaceutical companies and tumor groups.^7^ However, pCODR submissions have shown to be predominantly led by the pharmaceutical industry, with very few that are clinician led.^7^ The pCODR strives to evaluate oncology drug effectiveness and cost‐effectiveness in a timely and transparent manner.^7^ After an oncology drug indication receives a pCODR recommendation, it may then be considered eligible for the pan‐Canadian Pharmaceutical Alliance (pCPA) negotiation process.^8^ The pCPA’s purpose is to conduct joint public drug plan negotiations with manufacturers for drugs that are being considered for reimbursement in participating provincial, territorial, and federal programs.^8^ The pCPA uses the health technology assessment recommendation to help decide whether or not to enter into a negotiation for a drug.^8^ If the pCPA proceeds and successfully negotiates with a manufacturer, a letter of intent is issued.^8^ Participating drug plans are then able to make a final decision to reimburse a drug and subsequently enter into their own separate agreement with the drug manufacturer.^8^ These negotiation and reimbursement processes are intended to increase patient access to clinically effective treatments.^8^ However, it is presently unclear if these processes enable oncology drug indications with evidence of high clinical benefit to be reimbursed faster than those with evidence of low clinical benefit.

Thus, the aim of our study was to investigate if the time from pCODR recommendation to public reimbursement in Canada is associated with the degree of clinical benefit of oncology drug indications.

## 2 METHODS

### 2.1 Inclusion criteria

Oncology drug indications were identified using the pCODR website.^7^ All drug indications submitted from 13 July 2011 to 31 October 2018 were examined. Each included indication needed to have a NOC date, completed the pCODR review process, received a positive pCODR funding recommendation, and been funded by at least one province in Canada. In cases where the manufacturer resubmitted a drug indication, the most recent submission was used. If a drug indication was reimbursed publicly prior to pCODR recommendation in a province (i.e., had a negative time to public reimbursement), then that reimbursement time was excluded from our analyses.

### 2.2 Data extraction

Data were extracted independently by two reviewers (TJ and SD). Data discrepancies were resolved through consensus or a third reviewer (KC). The following variables were extracted: generic drug name, pCODR file number, cancer type, manufacturer submitted indication, route of administration, NOC date, submission date, initial recommendation date, final recommendation date, pCODR Expert Review Committee (pERC) final recommendation, and phase of trial cited by pCODR. Additionally, from the provincial funding summary the following variables were extracted: provincial funding status (funded, not funded, under negotiation with manufacturer, and under provincial consideration), provincial decision date, provincial funding date, provincial funding criteria, and revised decision date/funding criteria (if applicable).

Pivotal trials used by pCODR to evaluate the overall clinical benefit are stated and described in the pERC final recommendation document. We identified these trials and extracted them from the PubMed database. All primary and follow‐up publications of trials cited by pCODR that were published before the pCODR final recommendation date were included in the clinical benefit evaluation.

### 2.3 Clinical benefit evaluation

Two independent reviewers (LE and VN) scored all included trials using the American Society of Clinical Oncology Value Framework (ASCO‐VF) v2 and the European Society for Medical Oncology Magnitude of Clinical Benefit Scale (ESMO‐MCBS) v1.1.

The ASCO‐VF sums a drug's clinical benefit score, toxicity score, and any bonus points (e.g., quality of life (QoL) bonus) to yield a net health benefit (NHB).^9^, ^10^ According to the ASCO framework, if the experimental regimen is more toxic than the control regimen, the toxicity score is subtracted from the clinical benefit score.^9^, ^10^ If the experimental regimen is less toxic than the control regimen, the toxicity score is added to the clinical benefit score.^9^, ^10^ Thus, the NHB can theoretically have a negative value, however, it is rare to have a negative value for a positive clinical trial. ASCO classifies drugs with NHB < 45 as non‐substantial benefit and drugs with NHB ≥ 45 as substantial benefit.^11^

The ESMO‐MCBS assigns a preliminary grade based on the efficacy of the primary endpoint of a trial and a final ESMO‐MCBS grade after an evaluation of various scoring adjustments, such as QoL improvements and toxicities.^12^, ^13^ ESMO classifies ESMO‐MCBS scores < 4 as non‐substantial benefit and ≥4 as substantial benefit on a scale from 1 to 5.^11^

The ASCO and ESMO have reported their scores of some drug trials using transparent and clearly defined criteria. If an indication had previously been scored by ASCO and/or ESMO, the published score was used.^11^ Some published scores used data from follow‐up studies that were published after the pCODR final recommendation date. In this case, only the data available before the final recommendation date were used. If ASCO or ESMO scores were not publicly reported by ASCO or ESMO, then we derived those scores using the ASCO and ESMO frameworks in duplicate by two authors. If there were discrepancies between the authors’ scores, they were resolved through consensus and/or by a third reviewer (KC).

The ASCO‐VF and the ESMO‐MCBS each have certain scoring limitations. For instance, the ESMO‐MCBS allows the scoring of single‐arm trials, while the ASCO‐VF does not.^9^, ^10^, ^12^, ^13^ In addition, the ASCO‐VF allows the scoring of hematological trials, while the ESMO‐MCBS does not.^9^, ^10^, ^12^, ^13^ Furthermore, the ESMO‐MCBS allows the scoring of non‐inferiority trials, while the ASCO‐VF does not.^9^, ^10^, ^12^, ^13^ There were also cases where hematological trials (which cannot be scored by ESMO‐MCBS) had trial characteristics (design, endpoints, etc.) that did not allow them to be scored by ASCO‐VF either. For instance, one hematological trial evaluated the endpoints hematocrit control and reduction in spleen volume, which are not eligible for ASCO‐VF scoring.^9^, ^10^ Therefore, subsequent analysis of the time to reimbursement and clinical benefit using ASCO‐VF and ESMO‐MCBS only included drug indications that were scoreable by their respective frameworks.

### 2.4 Outcome

The primary outcome of this retrospective cohort study was the time it took, in months, for each province to reimburse each included drug indication from the date of pCODR recommendation. If some provinces decided not to publicly reimburse a drug indication, those provinces were not included for that indication.

### 2.5 Statistical analyses

The extracted data were analyzed using Spearman's correlation coefficient, univariable and multivariable linear regressions, and sensitivity analyses. Based on eligibility criteria, all included drug indications had received public funding, and therefore, had complete time to reimbursement data without any censoring. Thus, for our primary analysis, instead of using time‐to‐event analysis which is designed to handle censoring of time‐to‐event data, we elected to use mixed‐effects linear regression models, as we have complete data of reimbursement time without any censoring for the examined drugs. This allowed the regression coefficients to represent the association between potential explanatory variables and time to reimbursement in the natural time unit of months. Additionally, we conducted a sensitivity analysis using proportional hazards regression models to demonstrate robustness of the findings of associations.

Spearman's correlation coefficient was used to examine the correlation between clinical benefit (as per ASCO‐VF or ESMO‐MCBS) and time to public reimbursement from final pCODR recommendation of each indication for all provinces where the drug is publicly reimbursed.

In the univariable and multivariable analyses, clinical benefit was classified as substantial (ASCO ≥ 45, ESMO ≥ 4) or non‐substantial (ASCO < 45, ESMO < 4), as per the classification by the framework developers.^11^ Cancer indication was modeled as a random effect in the univariable and multivariable analyses in order to account for potential clustering of the time to reimbursement of the same indication in different provinces. Covariates used in the univariable and multivariable analyses included: pCODR recommendation with respect to cost‐effectiveness (recommends funding or recommends funding on the condition that cost‐effectiveness is improved), level of evidence (phase III or phase II), year of pCODR recommendation (2012–2014 or 2015–2018), province funding the drug indication, and cancer type. The covariate pCODR recommendation with respect to cost‐effectiveness was specifically chosen to assess whether drug indications that have already been deemed cost‐effective receive funding status faster than those that need to improve cost‐effectiveness. For the multivariable analyses, two separate models were constructed. The first used ASCO‐VF scores as the measure of clinical benefit and the second used ESMO‐MCBS scores.

Additionally, in the sensitivity analysis, ASCO‐VF and ESMO‐MCBS scores were modeled as continuous variables using raw scores. This was conducted to see if the multivariable results were robust. Statistical analyses were preformed using R (version 3.6.0).

## 3 RESULTS

### 3.1 Characteristics of identified studies

**FIGURE 1:**
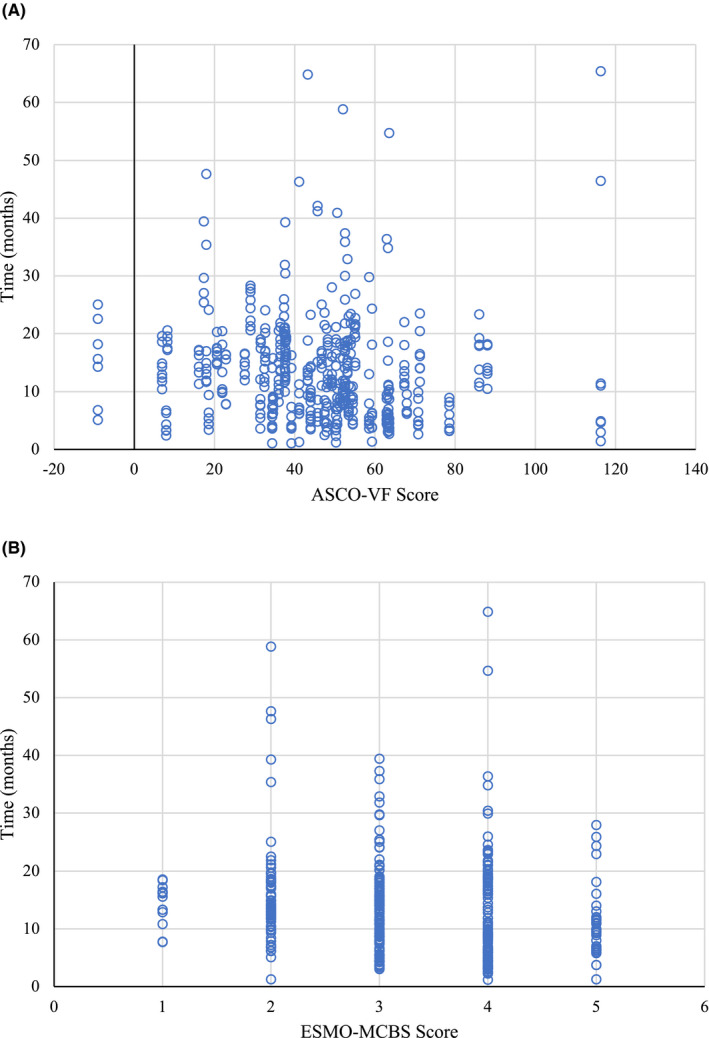
Time to public reimbursement from health technology assessment recommendation versus (A) raw ASCO‐VF score and (B) raw ESMO‐MCBS score. ASCO‐VF, American Society of Clinical Oncology Value Framework; ESMO‐MCBS, European Society for Medical Oncology Magnitude of Clinical Benefit Scale

Eighty‐four drug indications were identified (Table 1). Of the identified indications, 54 were solid tumors and 30 were hematologic. Sixty‐five ASCO‐VF v2 and 50 ESMO‐MCBS v1.1 scores were generated. The mean ASCO‐VF and ESMO‐MCBS scores were 44.9 (SD = 21.1) and 3.3 (SD = 1.0), respectively. The proportion of ASCO‐VF and ESMO‐MCBS scores that had substantial benefit was 52.3% and 44.0%, respectively. Additionally, the mean time to public reimbursement from pCODR recommendation was 13.2 months (SD = 9.3 months). The time to public reimbursement versus raw ASCO‐VF and ESMO‐MCBS scores are presented in Figure 1.

**TABLE 1:** Baseline characteristics and summary data of identified drug indications

Variable	Value
Distinct drug indications reviewed	105
Distinct drugs (chemical entities) reviewed	52
Indications that received provincial reimbursement (in at least one province)	84
Route of administration	
Oral	45
Intravenous	39
Year of pCODR recommendation	
2012	7
2013	16
2014	10
2015	17
2016	13
2017	12
2018	9
Tumor group	
Breast	5
Endocrine	2
Gastrointestinal	8
Genitourinary	6
Gynecology	2
Hematologic	30
Lung	13
Skin and melanoma	14
Other	4
ASCO‐VF score	
Mean	44.9
Median	46.7
Standard deviation	21.1
Minimum	−9
Maximum	116.3
ESMO‐MCBS score	
Mean	3.3
Median	3
Standard deviation	1.0
Minimum	1
Maximum	5
Time to provincial reimbursement from pCODR recommendation (months)	
Mean	13.2
Median	11.4
Standard deviation	9.3
Minimum	1.1
Maximum	65.4

Abbreviations: ASCO‐VF, American Society of Clinical Oncology Value Framework; ESMO‐MCBS, European Society for Medical Oncology Magnitude of Clinical Benefit Scale; NOC, Notice of Compliance; pCODR, pan‐Canadian Oncology Drug Review.

### 3.2 Spearman's correlation coefficient

Both ASCO‐VF and ESMO‐MCBS displayed a weak correlation with the time to public reimbursement, (*ρ* = −0.21, *p* < 0.0001) and (*ρ* = 0.24, *p* < 0.0001), respectively, suggesting a weak relationship between shorter time to reimbursement and drug indications that have higher clinical benefits.

### 3.3 Univariable analyses

The pCODR recommendation in regard to cost‐effectiveness, year of pCODR recommendation, and province were all associated with time to public reimbursement in univariable analyses. Earlier years (2012–2014) had shorter time to reimbursement (mean = 10.6) than later years (2015–2018) (mean = 15.3). ASCO‐VF scores, ESMO‐MCBS scores, level of evidence (phase III or phase II), and cancer type were not associated with reimbursement times (*p* = 0.12, *p* = 0.075, *p* = 0.87, and *p* = 0.11, respectively). The univariable results are presented in Table 2.

**TABLE 2:** Univariable analysis results

Variables	Frequency *n* (%)	Coefficient (months)	Mean (months)	Chi *p* value
ASCO‐VF score				
Substantial benefit (ASCO ≥ 45)	275 (55)	−2.0	12.7	0.12
Non‐substantial benefit (ASCO < 45)	225 (45)		14.7	
ESMO‐MCBS score				
Substantial benefit (ESMO ≥ 4)	181 (46)	−2.7	11.7	0.075
Non‐substantial benefit (ESMO < 4)	209 (54)		14.4	
pCODR recommendation				
Recommends funding	64 (13)	−4.1	9.2	0.019
Recommends funding if cost‐effectiveness is improved	436 (87)		13.5	
Level of evidence				
Phase III	485 (97)	0.25	13.7	0.87
Phase II	15 (3)		13.4	
Year of pCODR recommendation				
2012–2014	207 (41)	4.7	10.6	<0.0001
2015–2018	293 (59)		15.3	
Province				
1	58 (12)	0.73	12.0	<0.0001
2	58 (12)	(ref)	12.7	
3	64 (13)	−2.0	10.0	
4	63 (13)	−0.89	11.1	
5	59 (12)	−1.8	10.2	
6	56 (11)	3.4	15.4	
7	61 (12)	2.7	14.6	
8	53 (11)	4.4	16.3	
9	28 (6)	20	32.1	
Cancer type				
Breast	37 (7)	(ref)	14.7	0.11
Endocrine	13 (3)	1.7	16.3	
Gastrointestinal	54 (11)	3.5	18.3	
Genitourinary	52 (10)	−5.5	9.2	
Gynecology	16 (3)	1.6	16.3	
Hematologic	118 (24)	−0.89	13.8	
Lung	94 (19)	−2.8	11.9	
Skin and melanoma	92 (18)	−2.5	13.2	
Other	24 (5)	−1.5	13.2	

Abbreviations: ASCO‐VF, American Society of Clinical Oncology Value Framework; ESMO‐MCBS, European Society for Medical Oncology Magnitude of Clinical Benefit Scale; pCODR, pan‐Canadian Oncology Drug Review.

### 3.4 Multivariable analyses

**FIGURE 2:**
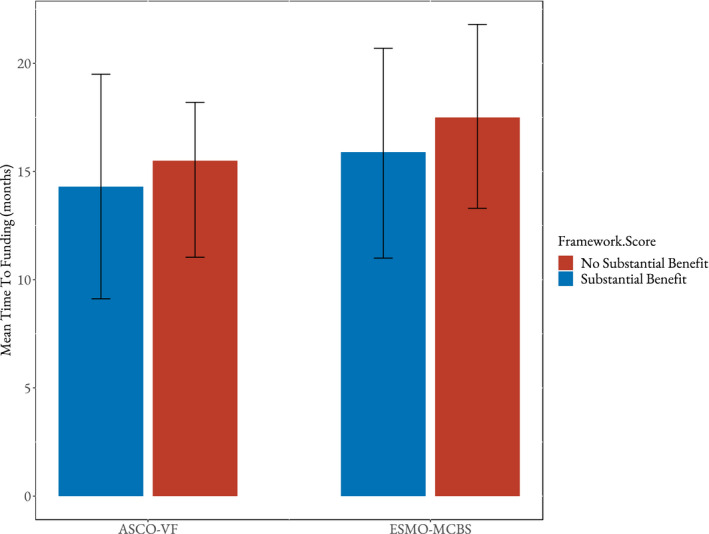
Mean time from health technology assessment recommendation to public reimbursement (substantial vs. non‐substantial benefit based on ASCO‐VF and ESMO‐MCBS). ASCO‐VF, American Society of Clinical Oncology Value Framework; ESMO‐MCBS, European Society for Medical Oncology Magnitude of Clinical Benefit Scale. Substantial benefit: ASCO ≥ 45, ESMO ≥ 4. Non‐substantial benefit: ASCO < 45, ESMO < 4

After adjusting for potential confounders, both ASCO‐VF and ESMO‐MCBS scores were not independently significantly associated with time to public reimbursement (*p* = 0.40 and *p* = 0.31, respectively). The mean times to reimbursement for ASCO‐VF substantial and non‐substantial scores were 14.3 months and 15.5 months, respectively (Figure 2). For substantial and non‐substantial ESMO‐MCBS scores, the mean times to reimbursement were 15.9 months and 17.5 months, respectively (Figure 2). The level of evidence (phase III or phase II) remained unassociated with reimbursement times in both ASCO and ESMO models (*p* = 0.82 and *p* = 0.87, respectively). Additionally, pCODR funding recommendation (in regard to cost‐effectiveness) was not associated with time to reimbursement in ASCO nor ESMO models (*p* = 0.76 and *p* = 0.078, respectively). Year of pCODR recommendation and province remained associated with time to public reimbursement in both models. Earlier years (2012–2014) had shorter time to reimbursement than later years (2015–2018) in both ASCO and ESMO models (*p* = 0.0044 and 0.0080, respectively). Additionally, cancer type remained significantly unassociated with time to public reimbursement in the ASCO model (*p* = 0.094) but was significantly associated in the ESMO model (*p* = 0.015). The multivariable results are reported in Table 3.

**TABLE 3:** Multivariable analysis results

Variables	ASCO‐VF model				ESMO‐MCBS model			
	Frequency *n* (%)	Coefficient (months)	Adjusted Mean (months)	Chi *p* value	Frequency *n* (%)	Coefficient (months)	Adjusted Mean (months)	Chi *p* value
Fixed effects								
Clinical benefit								
Substantial benefit	275 (55)	−1.2	14.3	0.40	181 (46)	−1.6	15.9	0.31
Non‐substantial benefit	225 (45)		15.5		209 (54)		17.5	
pCODR recommendation								
Recommends funding	64 (13)	−0.73	14.6	0.76	33 (9)	5.7	19.5	0.078
Recommends funding if cost‐effectiveness is improved	436 (87)		15.3		357 (91)		13.9	
Level of evidence								
Phase III	485 (97)	−0.91	14.5	0.82	359 (92)	−0.44	16.5	0.87
Phase II	15 (3)		15.4		31 (8)		16.9	
Year of pCODR recommendation								
2012–2014	207 (41)	4.4	12.7	0.0044	174 (45)	4.4	14.5	0.0080
2015–2018	293 (59)		17.1		216 (55)		18.9	
Province								
1	58 (12)	0.13	12.5	<0.0001	46 (12)	−0.71	13.8	<0.0001
2	58 (12)	(ref)	12.4		46 (12)	(ref)	14.5	
3	64 (13)	−2.5	9.9		49 (13)	−2.8	11.7	
4	63 (13)	−1.1	11.2		48 (12)	−1.2	13.3	
5	59 (12)	−2.2	10.2		45 (12)	−2.4	12.2	
6	56 (11)	3.2	15.5		43 (11)	3.2	17.7	
7	61 (12)	2.0	14.3		48 (12)	1.4	15.9	
8	53 (11)	2.0	14.4		42 (11)	1.6	16.2	
9	28 (6)	21	34.0		23 (6)	20.3	34.8	
Cancer type								
Breast	37 (7)	(ref)	17.0	0.094	37 (9)	(ref)	19.2	0.015
Endocrine	13 (3)	−0.41	16.6		13 (3)	−0.050	19.2	
Gastrointestinal	54 (11)	2.7	19.7		54 (14)	3.5	22.7	
Genitourinary	52 (10)	−6.6	10.4		52 (13)	−8.7	10.5	
Gynecology	16 (3)	−0.39	16.6		16 (4)	−0.6	18.6	
Hematologic	118 (24)	−1.3	15.7		NA	NA	NA	
Lung	94 (19)	−4.5	12.5		94 (24)	−3.9	15.3	
Skin and melanoma	92 (18)	−2.7	14.2		108 (28)	−2.9	16.3	
Other	24 (5)	−5.3	11.7		16 (4)	−7.52	11.7	

Abbreviations: ASCO‐VF, American Society of Clinical Oncology Value Framework; ESMO‐MCBS, European Society for Medical Oncology Magnitude of Clinical Benefit Scale; pCODR, pan‐Canadian Oncology Drug Review.

### 3.5 Sensitivity analyses

The sensitivity analyses modeled ASCO‐VF and ESMO‐MCBS scores as continuous values rather than a binary variable (substantial or non‐substantial benefit). Neither the ASCO‐VF scores nor ESMO‐MCBS scores were associated with reimbursement times (*p* = 0.78 and *p* = 0.33, respectively).

One province had mean time to reimbursement much larger than the overall average which may be viewed as an outlier. This province was excluded as an exploratory analysis. After adjustment, province continued to have a significant association with reimbursement times in all models. In addition, ASCO‐VF and ESMO‐MCBS scores remained unassociated with reimbursement times (*p* = 0.32 and *p* = 0.27, respectively).

Furthermore, sensitivity analyses using proportional hazard regression models were conducted to determine the robustness of the findings of association. Both ASCO‐VF and ESMO‐MCBS scores remained unassociated with reimbursement times (*p* = 0.73 and *p* = 0.27, respectively).

## 4 DISCUSSION

Our study found that oncology drug indications with high clinical benefit (as per ASCO‐VF and ESMO‐MCBS) were not significantly associated with shorter time to public reimbursement. The pCODR recommendation (in regard to cost‐effectiveness) and level of evidence (phase III or phase II) were similarly not associated with public reimbursement times. The attributes most associated with reimbursement times were the year of pCODR recommendation, the province where the reimbursement decision was made, and the type of cancer.

The drug review process in Canada appears to follow a “first in, first out” approach.^14^ Drug indications also appear to be reimbursed publicly in the order they were reviewed with no regard to their clinical benefit. This current approach may be attributed to the absence of an explicit prioritization process.^14^ First, the pCODR gives recommendation about which drug indications to reimburse but does not explicitly state which ones have relatively higher clinical benefit.^14^ Second, the pCODR framework states which criteria to consider in the decision‐making process but does not deliver guidance to the pCPA or public drug plans on the relative rankings, weights, or thresholds assigned to each criterion.^15^ Third, the pCPA and provincial/territorial decision‐makers do not have a transparent system of drug prioritization, which makes it difficult to decide which drug should enter the negotiation and reimbursement process first.^15^ Therefore, the current approach does not specifically foster reduced reimbursement times for drug indications of higher clinical benefit.

Comparable results have been found when examining oncology drugs that have received regulatory approval by the FDA.^16^, ^17^, ^18^ A study found that 38% of FDA‐approved oncology drugs were approved based on response rate (RR) and 34% were approved based on progression‐free survival (PFS).^16^ This led to an estimated reduction in development time of 19 months and 11 months for trials using RR and PFS, respectively.^16^ While FDA approval timelines may have been improved, an analysis of 36 oncology drugs approved based on surrogate endpoints found that only 5 later displayed an improvement in overall survival (OS).^17^ Furthermore, Gyawali et al.^18^ found that, out of 93 oncology drug accelerated approvals, only 20% demonstrated an improvement in OS in confirmatory trials. The rest of the accelerated approvals were found to conduct confirmatory studies using surrogate measures, with many using the same surrogate that was used in the preapproval trial.^18^ Therefore, through the use of surrogate endpoints and accelerated pathways, the FDA appears to be prioritizing speed but consequently may be overlooking clinical benefit.^16^, ^17^, ^18^ Regulatory approval in the United States and post‐pCODR reimbursement processes in Canada may both benefit from the use of more explicit criteria of the magnitude of clinical benefit.

A low correlation between time to access and clinical benefit of oncology drugs has additionally been observed in European healthcare systems.^19^, ^20^ Ferrario et al.^19^ found no correlation with ESMO‐MCBS scores when examining the time from European Medicines Agency (EMA) marketing authorization to first use of oncology drugs in Belgium, Estonia, Scotland, and Sweden. Furthermore, Janzic et al.^20^ found that the time from EMA marketing authorization to national reimbursement approval in Slovenia was equally long for oncology drugs with or without substantial clinical benefit, as measured by the ESMO‐MCBS. These studies assessed a heterogenous range of the time period from regulatory approval to first use or reimbursement, which might be influenced by many processes or factors. Our study specifically focuses on the time it took for manufacturers and public payers to negotiate and for payers to reach a decision for reimbursement of oncology drug indications after a positive health technology assessment recommendation. Thus, our study provides a unique perspective about the factors associated with the time for public oncology drug reimbursement decision‐making and provides further evidence that clinical benefit does not appear to be a main factor in relation to the speed of reimbursement decisions.

Implementing a transparent system of prioritization is one strategy to ensure timeliness is proportional to the clinical benefit of drugs. Recently, multicriteria decision analysis (MCDA) has been gaining traction in health care and oncology decision‐making.^21^ Indeed, the International Society for Pharmacoeconomics and Outcomes Research (ISPOR) has published guidelines for implementing MCDA into health care decision‐making.^22^, ^23^ MCDA helps to guide the prioritization process through the use of a rating tool.^24^, ^25^ A rating tool is developed by a group of relevant stakeholders and used to assess each treatment option against a pre‐defined list of criteria.^24^, ^25^ The three main components of a rating tool include a relevant list of criteria, a weight assigned to each criteria, and a rating scale for each criteria.^25^ The results of the rating tool are then used to lead deliberative discussion between stakeholders and assist them in setting funding priorities.^26^, ^27^ Previously, MCDA methods have been used in South Africa to help make decisions on coverage of cervical cancer screening and in Thailand to guide decisions for a health benefit package.^28^, ^29^ In addition, the United Kingdom has developed an MCDA‐based framework to help assess the value of orphan drugs and frame a more structured discussion between decision‐makers in the reimbursement process.^30^ Furthermore, Ezeife et al.^21^ have developed an MCDA‐based drug assessment framework (DAF) specifically for oncology drugs. With provincial budgetary constraints, they suggested using the DAF to help public payers identify and prioritize the highest impact drugs of those that have received a positive pCODR recommendation.^21^

An additional consideration in the examination of reimbursement times from pCODR recommendation is the increasing number of submissions to pCODR. In our study, we observed that there was a significant increase in the time to reimbursement from pCODR recommendation in more recent years (2015–2018) compared to earlier years (2012–2014). This may be partly due to the fact that there were more submissions to pCODR and more positive recommendations in recent years. Ultimately, this can place greater strain on the pCPA and public payers who must enter negotiations for these drug indications. Therefore, if there was no corresponding increase in pCPA staff, they would be unable to complete negotiations in a timely manner leading to longer times to public reimbursement. In the case that pCPA staff has not increased, prioritization of drugs with greater clinical benefit would be even more important to ensure that at least the most effective drugs are being reimbursed in a timely manner.

Our study has some notable limitations. First, we used ASCO‐VF and ESMO‐MCBS scores as a measure of clinical benefit. These frameworks have been developed by the two largest oncology professional organizations to systematically assess the clinical benefit of oncology treatments. Nevertheless, we recognize that there are other tools in literature, and that no single tool is regarded as perfect or the gold standard for measuring clinical benefit.^31^ Therefore, any limitations of the tools we chose to use will inherently be limitations of our study. In addition, our analysis could be limited by the potential lack of complete dates for funding status, which depends on the completeness of the information posted publicly by the pCODR website. When a drug indication was still being considered for publicly reimbursement at the time when we conducted the study, it was listed in the pCODR website as “under provincial consideration” and thus could not be included in our analysis.

## 5 CONCLUSION

Oncology drug indications of higher clinical benefit do not seem to be associated with a reduced time to public reimbursement in Canada. It may be beneficial to consider implementing a transparent system of prioritization that would ensure Canadians have the quickest access to treatments with the potential for greatest clinical benefits.

## CONFLICT OF INTEREST

None.

## AUTHOR CONTRIBUTIONS

Sasha Thomson: Investigation, Visualization, Project administration, Writing‐original draft preparation, and Writing‐review and editing; Louis Everest: Investigation, Visualization, Formal analysis, Writing‐original draft preparation, and Writing‐review and editing; Noah Witzke: Investigation, Visualization, and Writing‐review and editing; Tina Jiao: Investigation and Writing‐reviewing and editing; Seanthel Delos Santos: Investigation and Writing‐reviewing and editing; Vivian Nguyen: Investigation and Writing‐reviewing and editing; Matthew C. Cheung: Writing‐reviewing and editing; Kelvin K.W. Chan: Conceptualization, Methodology, Supervision, and Writing‐reviewing and editing.

## ETHICAL APPROVAL

Ethical approval was not required for this study because it was based on publicly available data and did not involve individual patient data collection or analysis.

## Data Availability

The data that support the findings of this study were derived from the following resources available in the public domain: pCODR (https://www.cadth.ca), ASCO‐VF v2 (https://doi.org/10.1200/JCO.2016.68.2518), ESMO‐MCBS v1.1 (https://doi.org/10.1093/annonc/mdx310), and Cherny et al. (https://doi.org/10.1200/JCO.18.00729).
